# Selection of suitable endogenous reference genes for qPCR in kidney and hypothalamus of rats under testosterone influence

**DOI:** 10.1371/journal.pone.0176368

**Published:** 2017-06-07

**Authors:** Khadijeh Gholami, Su Yi Loh, Naguib Salleh, Sau Kuen Lam, See Ziau Hoe

**Affiliations:** 1 Division of Human Biology, School of Medicine, International Medical University, Kuala Lumpur, Malaysia; 2 Department of Physiology, Faculty of Medicine, University of Malaya, Kuala Lumpur, Malaysia; Northwestern University Feinberg School of Medicine, UNITED STATES

## Abstract

Real-time quantitative PCR (qPCR) is the most reliable and accurate technique for analyses of gene expression. Endogenous reference genes are being used to normalize qPCR data even though their expression may vary under different conditions and in different tissues. Nonetheless, verification of expression of reference genes in selected studied tissue is essential in order to accurately assess the level of expression of target genes of interest. Therefore, in this study, we attempted to examine six commonly used reference genes in order to identify the gene being expressed most constantly under the influence of testosterone in the kidneys and hypothalamus. The reference genes include glyceraldehyde-3-phosphate dehydrogenase (GAPDH), actin beta (ACTB), beta-2 microglobulin (B2m), hypoxanthine phosphoribosyltransferase 1 (HPRT), peptidylprolylisomerase A (Ppia) and hydroxymethylbilane synthase (Hmbs). The cycle threshold (Ct) value for each gene was determined and data obtained were analyzed using the software programs NormFinder, geNorm, BestKeeper, and rank aggregation. Results showed that Hmbs and Ppia genes were the most stably expressed in the hypothalamus. Meanwhile, in kidneys, Hmbs and GAPDH appeared to be the most constant genes. In conclusion, variations in expression levels of reference genes occur in kidneys and hypothalamus under similar conditions; thus, it is important to verify reference gene levels in these tissues prior to commencing any studies.

## Introduction

Real time Quantitative PCR (qPCR) is a sensitive technique frequently used to evaluate gene expression whereby data are collected throughout the PCR amplification process [1]. This method combines both amplification and detection of the PCR products into a single step procedure; thus it can produce accurate data and does not necessitate post-amplification manipulation [1]. In addition, this technique is able to detect small differences in gene expression between samples while requiring less mRNA templates in comparison to other methods. Moreover, this specific method with large dynamic range of RNA quantification has the capacity for high throughput of data [2].

In qPCR, as in other procedures, correct normalization of the method is imperative in order to obtain accurate and trustworthy results. Different normalization strategies have been employed to reduce variability in the qPCR amplification process including internal reference genes (RGs), normalization to initial amount of material and use of external control nucleotides [3–5]. Applying the use of internal RGs is the most accepted method since expression and amplification of both reference and target genes occur under the same conditions despite different sequences [2]. The main criteria for a suitable RG are that its expression is unaffected by experimental conditions, and it shows minimal variability between different tissues under different physiological conditions [6].

Housekeeping genes were known to be ideal candidates as RGs, since they are involved in the essential processes for cell survival, hence their expression is supposed to be stable in various conditions [6,7]. However, housekeeping genes may also participate in different metabolic processes and may not be ideal RGs for all conditions [6].

Genes such as glyceraldehyde-3-phosphate dehydrogenase (GAPDH), actin beta (ACTB) and 18S ribosomal RNA (18S) have been widely used as RGs in northern blotting and RT-PCR, based on the assumption that their expression levels are constant under different conditions [3,6,8,9]. However, there is no universally applicable gene with invariant expression [10,11], and the expression of historical RGs has been reported to change in numerous tissues and in different study protocols [2,3,5,9,12–14].

Previous studies have shown that expression of various housekeeping genes were altered by hypoxia [15], transplantation [16], and sex steroids [8,9,17]. The expression of some housekeeping genes [9,17], such as tubulin, cyclophilin, tyrosine aminotransferase, ACTB, GAPDH and 18S in the liver of intact female and male rats, was shown to be sex-dependent as their levels of expression were different in both genders [18]. In a study by Cai *et al* [19] ACTB was used as a RGs following castration in pig, whilst in another study in aging men Polymerase (RNA) II Subunit A (POLR2A), Importin 8 (IPO8) and Ppia were identified as suitable genes for data analysis in biopsies of skeletal muscle following testosterone therapy [20]. There was no study to report of expression of housekeeping genes under the influence of testosterone in rats. Therefore, in this study, selection of suitable RGs in the kidney and hypothalamus of rats under the effect of testosterone was carried out as part of the preliminary studies for future experiments. The reasons to choose these tissues were: firstly, regulation of blood pressure is associated with extracellular fluid homeostasis and Na^+^ content; furthermore, the kidney is the principle organ for regulation of Na^+^ and body fluid which control blood pressure [21]. Secondly, behavioral and electrophysiological studies have identified multiple hypothalamic nuclei that respond to angiotensin II [22], that is involved in the control of blood pressure. On the other hand, in addition to age, genetics and environmental factors such as poor diet, lack of exercise, and increased body weight, gender is another factor that is involved in the regulation of blood pressure [21]. Thirdly, it has been reported that the incidence of high blood pressure in males is higher than in females [23–25], particularly the prevalence of hypertension in younger men is higher than the female counterparts [26]. Indeed animal studies showed that testosterone increased blood pressure in salt-loaded ovariectomized rats [27]. In addition, androgen enhances arterial pressure by causing hypertensive shift in the pressure-natriuresis relationship via direct effects on proximal tubular reabsorption in kidneys or activation of renin-angiotensin system [28], resulting in sodium retention [29] and therefore, increases blood pressure. On the other hand, testosterone influences electrolyte excretion in an androgen receptor dependent manner [30]. Hence, similar network of genes might be responsible for regulation of electrolyte and blood pressure in both tissue. This study, therefore, focused on 6 different commonly used RGs that were chosen from different functional classes to reduce the chance that genes might be co-regulated. Subsequently different statistical applets such as geNorm [31], NormFinder [32] and Bestkeeper [4] were used to analyze the data and evaluate the most stable gene in the tested conditions.

## Material and methods

### Treatment of animals

Eight weeks old male Sprague-Dawley rats (n = 16), weighing 220± 30g were obtained from the Animal Experimental Unit, Faculty of Medicine, University of Malaya. Animals were housed in a clean and well-ventilated environment of 12:12 hours light: dark cycle and temperature of 24°C. All procedures were carried out in strict accordance with the recommendations in the Guide from the Institutional Animal Care and Use Committee (IACUC), Faculty of Medicine, University of Malaya. The protocol was approved by the Committee on the Ethics of Animal Experiments of the University of Malaya (Ethics Number: 2014-05-06/physio/R/NS). All surgery was performed under ketamine (80mg/kg) and xylazine (8mg/kg) anesthesia (i.p injection) and efforts were made to minimize their suffering. Kombitrim (1 mg/kg/day, for 3 days, i.m injection) antibiotic was given to all animal in order to prevent post-surgical infection, while meloxicam (1mg/kg/day, 3days, s.c injection) was used as painkiller after surgery.

Sham-operation and orchidectomy (ORX) were performed 14 days prior to the start of the experiments. The rats were divided into 4 different groups (n = 4 per group): sham-operated, ORX, ORX with 125μg/kg/day or 250μg/kg/day testosterone (Sigma, USA) treatments. Testosterone (T) was given via subcutaneous injection for 7 days (once a day) continuously at 9 am. Sham-operated and ORX groups received vehicle peanut oil throughout the study. At the end of the treatment, rats were decapitated and hypothalamus and kidney were collected. To collect the hypothalamus, the brain was first separated from the skull, rinsed in cold RNAse-free phosphate-buffered saline, and placed ventral side up in a petri dish set in ice. With the naked eye and by using the optic chiasma as a landmark, a pair of curved forceps were used to easily remove the fresh hypothalamus from the ventral surface of the brain and, gently spooned out. There was a depression where the hypothalamus was excised. Since brain was not frozen the hypothalamus was easily separated. Next step was to remove the kidneys. To remove the kidneys, the abdomen was cut and the intestines were moved to reach the kidneys, which were separated from the vessels and the overlying adrenal gland was excised. The kidneys were washed in RNAse-free phosphate-buffered saline. Following tissue harvesting, specimens were snap frozen by using liquid nitrogen. All samples were then stored in -80°C for further RNA extraction.

### RNA extraction, quantification and cDNA synthesis

RNA extraction was performed by using Macherey-Nagel NucleoSpin RNA kit (Düren, Germany) following the manufacturer’s guidelines. Briefly, after tissue homogenization, RNA was purified by silica membrane, followed by washing with rDNase solution to eliminate any DNA contaminants. RNA was then eluted with RNase free water. The concentration of extracted RNA was assessed by Nano drop (Thermo Scientific, USA), and RNA purity was assessed by the ratio of absorbance at 260nm to 280nm and 260nm to 230nm in which a good quality RNA will have a ratio of A260/280 > 1.8 and A260/230 > 1.7 [33]. The integrity of RNA was evaluated using agarose gel electrophoresis. cDNA synthesis was then carried out by using BioradiScript Reverse Transcription Supermix for RT-qPCR (California, USA) according to the manufacturer’s manual, where 50 ng total RNA was used in a reaction volume of 10 ul.

### Real time quantitative PCR (qPCR)

TaqMan-FAM labeled assay (including primer and probe) for each RGs was purchased from Applied Biosystems (USA). The RGs of different functional classes used in this study are stated in Table 1. A 10 ul reaction mixture composed of 5 ul master mix (fast universal master mix, Applied Biosystem, USA), 2.9 ul RNase free water, 0.1 ul TaqMan assay, and 2 ul cDNA was prepared. qPCR amplification was carried out by using ABI Step One Plus (Applied Biosystems, USA) with the following running profiles: 50°C for 2 minutes, 95°C for 10 minutes followed by 40 cycles of amplification (95°C for 15 s and 60°C for 1 min). Four biological replicates and 3 technical replicates were used for each gene. Two negative controls were used to verify the qPCR results. One was non-template control (NTC) which served as a general control for extraneous nucleic acid contamination. The other one was no-RT control which omitted reverse transcriptase, and assessed the amount of DNA contamination in RNA preparation.

**Table 1:** Description and efficiency of six reference genes, used for qPCR.

Gene ID	Gene Function	NCBI reference	Assay ID	Amplicon Size(bp^†^)	Efficiency (%)
**Hmbs**	Involve in heme biosynthetic pathway	NM_013168.2	Rn00565886-m1*	99	94.54
**Ppia**	Accelerate the folding of proteins	NM_017101.1	Rn00690933-m1*	149	98.84
**B2m**	Beta-chain of major histocompatibility complex class I molecules	NM_012512.2	Rn00560865-m1*	58	103.54
**GAPDH**	Glycolytic enzyme, DNA repair, transcriptional regulation	NM_017008.3	Rn01775763-g1#	174	93.7
**Hprt**	Purine synthesis in salvage pathway	NM_012583.2	Rn01527840-m1*	64	106.76
**ACTB**	Cytoskeletal structural protein	NM_012583.2	Rn01527840-m1*	64	102.21

Acceptable range of efficiency is 90%-110%, which indicates the amount of amplification in each cycle.

* m: in the assay name indicates that primer span exon-exon and do not amplify the genomic DNA

^#^ g indicates that an assay may detect genomic DNA. The assay primers and probe may also be within a single exon

^†^: bp: base pair

The PCR efficiency (E) of each primer pair (Table 1) was tested with serial dilutions of cDNA in triplicate and was presented in percentage (%) according to the following equation: Efficiency = 10^(-1/slope)^ -1 [34].

### Data analysis

In order to find out the constant RG for qPCR, different statistical algorithms were used to calculate the stability of candidate RGs, first, GenEx (MultiD, Gothenburg, Sweden), which includes geNorm [31] and NormFinder [32] algorithm, and second, BestKeeper [4], a Microsoft Excel based algorithm.

In order to compare the transcription levels of different genes, their cycle threshold (Ct) [35] values were compared directly. A Ct value is the number of cycles at which fluoresces levels exceed the background level, and it is inversely correlated with the amount of nucleic acid in the reaction [36]. In this study, Ct values were calculated by ABI Step One Plus^™^ machine, and the raw Ct values were used for data analysis. There was no amplification in negative controls as indicated by “undetermined Ct value”, during data collection and this implied that primers were specific; and there was not nucleic acid contamination.

NormFinder was first described by Anderson *et al* [32], and it measures the stability of RGs, and ranks them according to their expression stabilities in a given set of samples and experimental design. It also estimates overall expression variations of the candidate normalization gene, and the variation among samples (control versus other groups). Therefore, the most stable gene is defined by the lower stability value.

GeNorm from GenEX software uses the same data as NormFinder, but all samples are treated as being from a single population, which means, it does not consider the effect of treatment. GeNorm sequentially eliminates the genes that show the highest variation relative to all the other genes based on pair expression value in all study samples and provides a measure of gene expression stability called the M-value that has a cut-off point of variability whereby any genes with M > 1.5 are considered as unstable. Thus, the lowest M-value indicates the most stable RG [31]. Since geNorm relies on pair wise comparison, it results in a combination of the two most stable genes [37].

Bestkeeper is a Microsoft Excel-based program that identifies the optimal RG by application of pair-wise co-relational analysis of all pairs of candidate genes via calculation of geometric mean of best choice [4]. This program produces standard deviation, coefficient of correlation (r) and “BestKeeper index” which are a series of descriptive statistics. In this method any genes with standard deviations greater than one are excluded from further analysis.

Application of different software for analysis of gene expression resulted in inconsistencies among various methods, which is due to distinct statistical algorithms in each one [38]. To overcome these discrepancies, therefore, the stability measurements of the chosen genes obtained from these software were combined to create a consensus rank of the genes by using Rank Aggreg package with R programming language [39]. Rank aggregation techniques offer a general and flexible frame to combine several-ordered list in a proper efficient manner. RankAggreg package is available through CRAN http://cran.r-project.org/web/packages/RankAggreg/. Weighted rank aggregation of ordered list was performed using the Brute Force approach, by generating all possible order lists and finding the list with minimum value of objective function. Spearman footrule distance are applied in Brute aggregation. It is nothing more than the summation of the absolute differences between the ranks of all unique elements from both ordered lists combined. It is rather a very intuitive metric for comparing two ordered lists of arbitrary length. The smaller the value of the metric, the more similar the lists [39]. The rank aggregations were conducted with R software version 3.2.

## Results

### Amplification efficiency

Amplification efficiencies of 6 different genes were measured following qPCR with 5 data points’ serial dilutions of each gene. Efficiencies between 90% and 110% indicated a robust and reproducible assay. The efficiencies of these genes are presented in Table 1.

### Validation of potential reference genes

**Fig 1:**
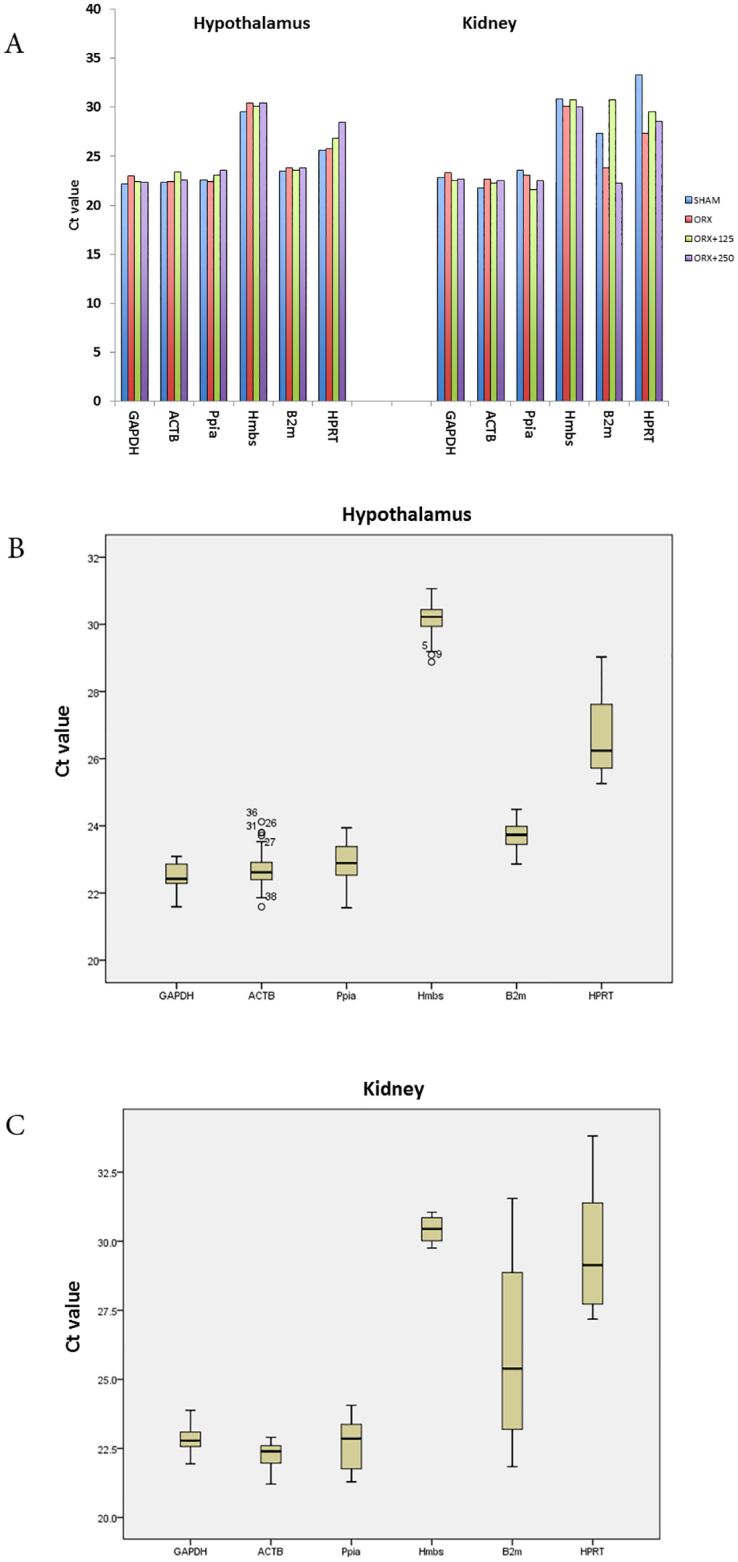
(A) Distribution of cycle threshold of 6 different genes in hypothalamus (S1 Table) and kidney (S2 Table); 4 biological and 3 technical replicates were used. Mean Ct value of each gene in all treated groups were described. Lower level of Ct value implies higher expression level as for GAPDH, ACTB, Ppia in both tissue and B2m only in hypothalamus. (B, C) Box plot graphs of Ct values are shown as a square across the box is depicted as the median. The box indicates the 25th and 75th percentiles and the whiskers caps represent the maximum and minimum values.

Results showed that intra-group variation of HPRT was higher than that of the other genes in the hypothalamus, which indicates HPRT might not be a suitable RG in the hypothalamus under these experimental conditions. In the kidney, variations in expression of B2m and HPRT were high (Fig 1), and in spite of higher Ct value of Hmbs, it was found to be more consistent across different treated groups in both tissues.

### Determination of stability of reference gene by GenEx

According to NormFinder software, lower stability expression value of a gene implies a more stable gene, and it is based on variance estimation approach. NormFinder ranked candidate genes according to their expression stability under given conditions [32]. The results showed that in the hypothalamus, Hmbs and Ppia were the most stable genes while in the kidney, Hmbs and GAPDH genes were the most stable (Table 2). Thus Hmbs appears to be an optimal RG in both tissues. However, the stability levels of other genes varied between the two tissues.

**Table 2:** Shows the expressional stability of selected reference genes, using NormFinder in hypothalamus and kidney. NormFinder algorithm is based on model-based approach to calculate overall reference gene stability. Gene with lower standard deviation (SD) is consider the most stable gene. In both tissues Hmbs is the most stable gene while in hypothalamus HPRT and in kidney B2m is the least stable one.

Hypothalamus	SD	Kidney	SD
Hmbs	0.31	Hmbs	0.34
Ppia	0.37	GAPDH	0.89
B2m	0.43	ACTB	1.07
GAPDH	0.55	Ppia	1.08
ACTB	0.55	HPRT	1.86
HPRT	1.04	B2m	3.21

**Fig 2:**
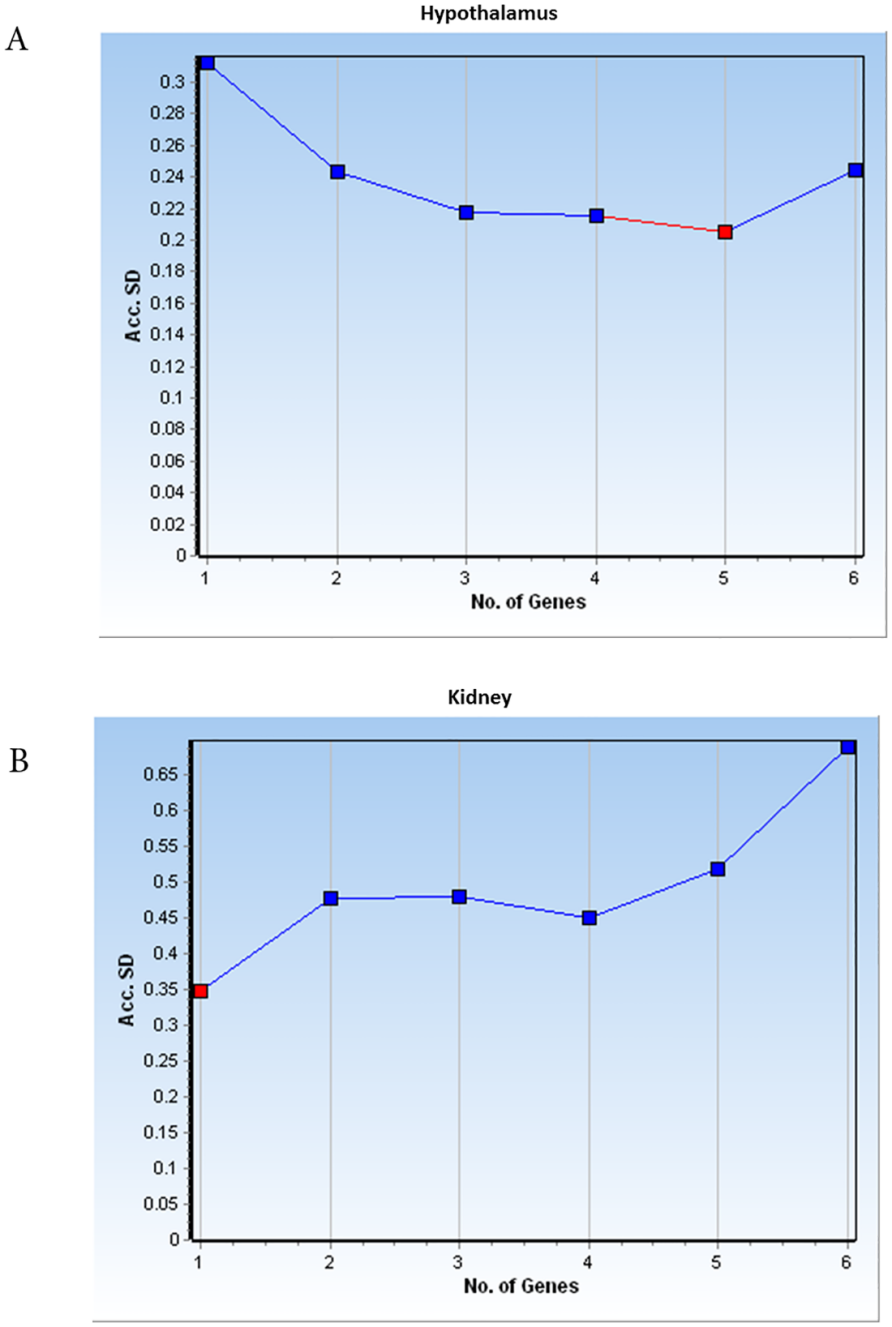
Presents number of optimal reference gene in hypothalamus (A) and kidney (B) which defined by NormFinder, whereby the lower value of accumulated standard deviation (Acc.SD), identified by red color, indicates the optimal number of reference genes.

Application of more than one RG for optimal analysis of qPCR was previously suggested in order to reduce the errors that may result from using only one gene [5,6,40]. This is in agreement with the introduction of two genes by geNorm and NormFinder. NormFinder defines the number of optimal RGs by calculating the Acc.SD. In this study, data analysis by NormFinder indicated application of one gene in the hypothalamus resulted in higher Acc.SD (0.3), whereas adding second gene reduces the Acc.SD (0.24). Inclusion of third to fifth RGs slightly reduces Acc.SD, which showed that using only one gene for analysis of qPCR data, under the influence of testosterone in hypothalamus, is not an appropriate candidate (Fig 2A). In the kidney (Fig 2B), however, using only one RG resulted in the lowest Acc.SD (0.35) and addition of second and third genes enhanced Acc.SD (Acc.SD = 0.45). Therefore, although one RG in kidney might be sufficient; and since inclusion of additional RGs requires more time and cost, at least two genes are adequate for normalization [41].

Therefore, in both tissues, two genes are sufficient to reach reliable normalization. Although the use of at least two RGs has been suggested, it is important to consider that, if a large number of RGs are used, random variation among the genes partially cancels the reduction in SD in addition to raising the cost of the study.

**Fig 3:**
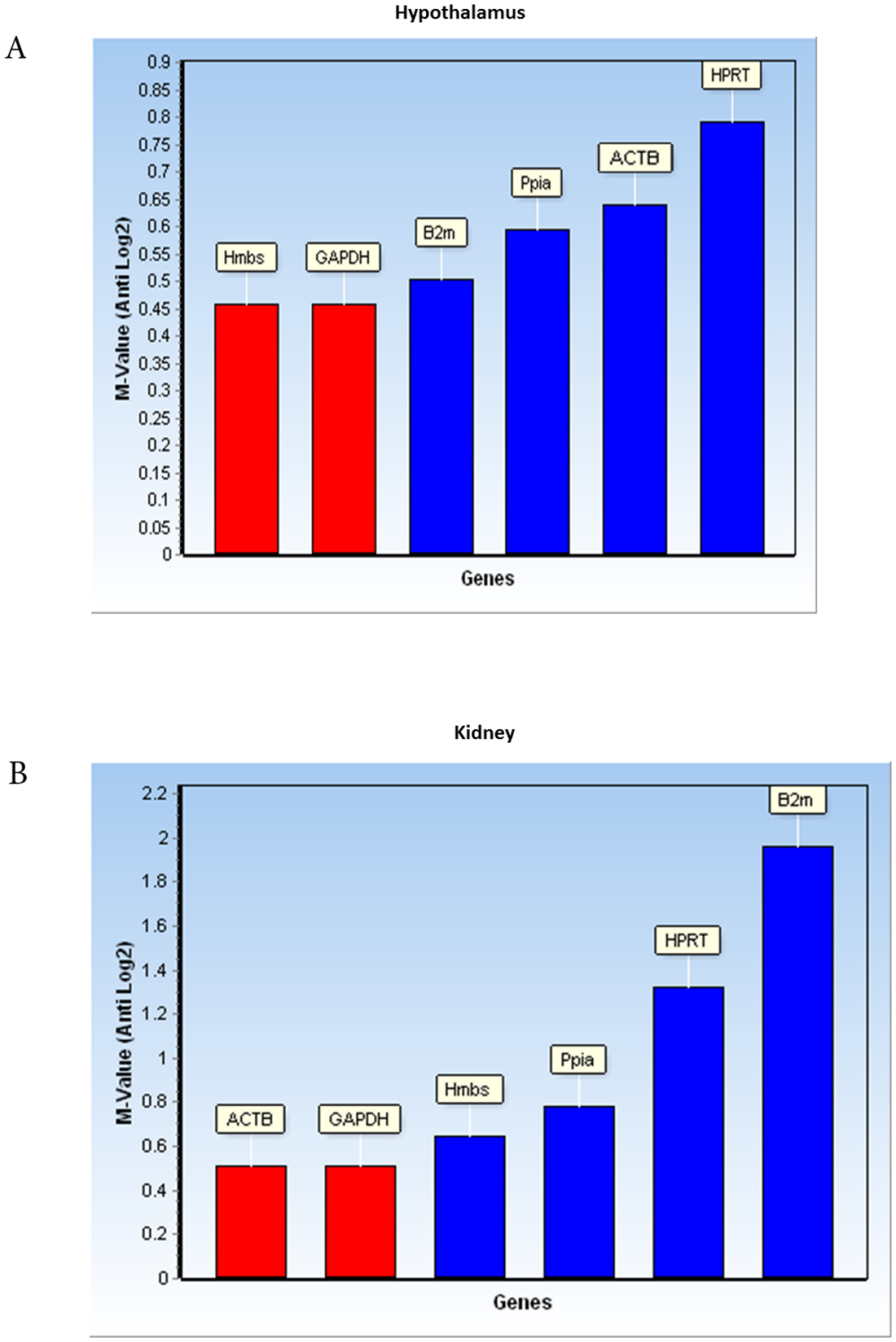
Gene stability value in hypothalamus (A) and kidney (B) is estimated by geNorm. The calculation is based on pairwise comparison between gene of choice and all other tested genes. M-value>1.5 will be eliminated from pairwise analysis.

GeNorm [31], on the other hand, defines the gene stability based on M-values, in which the lowest M-value denotes the most stable gene. According to geNorm analysis, the two suitable genes in the hypothalamus were Hmbs and GAPDH; while in the kidney, GAPDH and ACTB were the best candidates. The M-values of different genes in the hypothalamus and kidney are presented in Fig 3. Results showed that the M-values of all 6 genes in the hypothalamus were less than 1.5; thus any of these genes might be potential RGs for normalization of qPCR data under the influence of testosterone. However in the kidney B2m, with M-values >1.5, was not suitable RG for qPCR normalization.

### Determination of stability of reference gene by BestKeeper

BestKeeper identifies the optimal RG via calculation of coefficients of correlation (r) with geometric mean of all genes (the BestKeeper index) [4]. After calculation of BestKeeper index, standard deviation was calculated and those genes with SD > 1 were removed from further analysis. At the end, coefficient correlation (r) was calculated and genes with coefficient correlation closer to 1 were consider as appropriate gene (Table 3). In this method Ppia was the best gene in the hypothalamus, and Hmbs was the most stable gene in the kidney.

**Table 3:** Suitable reference genes for both hypothalamus and kidney. Shows the suitable reference genes for both hypothalamus and kidney were Ppia& Hmbs.

Gene (Hypothalamus)	coeff. of corr. [r]	Gene (Kidney)	coeff. of corr. [r]
Ppia	0.74	Hmbs	0.91
Hmbs	0.66	Ppia	0.12
ACTB	0.46	GAPDH	-0.24
B2m	0.38	ACTB	-0.62
GAPDH	0.10		

### Optimal list of genes by rank aggregation

In view of the different findings given by 3 analytical programs, the weighted rank aggregation via brute force algorithm with minimum Spearman footrule distance (conducted by R programming language, version 3.2) was used to combine the lists from GenEx and BestKeeper to a consensus rank of genes [14], and R script for rank aggregation is presented in S1 File. The M-values (from GeNorm), variability measurements (from NormFinder) and coefficients of correlation (from BestKeeper) were used as a weight in the aggregation process. As shown by the results presented in Table 4, Hmbs and Ppia in hypothalamus, and Hmbs and GAPDH in kidney, were identified as the most stable genes and were recommended as RGs for qPCR analysis under the effect of testosterone.

**Table 4:** Presents the final optimal list of genes in hypothalamus and kidney.

Hypothalamus				Kidney			
geNorm	NormFinder	BestKeeper	rank aggregation	geNorm	NormFinder	BestKeeper	rank aggregation
Hmbs	Hmbs	Ppia	Ppia	ACTB	Hmbs	Hmbs	Hmbs
GAPDH	Ppia	Hmbs	Hmbs	GAPDH	GAPDH	Ppia	GAPDH
B2m	B2m	ACTB	B2m	Hmbs	ACTB	GAPDH	Ppia
Ppia	GAPDH	B2m	GAPDH	Ppia	Ppia	ACTB	ACTB
ACTB	ACTB	GAPDH	ACTB	HPRT	HPRT		HPRT
HPRT	HPRT		HPRT	B2m	B2m		B2m

The final list is based on the results of geNorm, NormFinder and BestKeeper, which was ranked by rank aggregation.

## Discussion

Employing suitable normalization method during qPCR data analysis is essential in order to obtain a reliable quantification of the expressed genes. The most common strategy is the application of endogenous RGs, and hence selection of optimal RGs, expression of which remains stable across different samples is crucial [9]. In this study, the expression stabilities of six commonly used RGs in the hypothalamus and kidney under the influence of testosterone were analyzed by statistically validated program such as NormFinder, geNorm and BestKeeper; which were then followed by weighted rank aggregation to define the suitable genes from different algorithms.

According to the results, Hmbs was the most stably expressed gene in the hypothalamus as indicated by 3 different softwares, geNorm (Fig 3), NormFinder (Table 2) and BestKeeper (Table 3). On the other hand, HPRT was identified as the least stably expressed gene in the tested conditions by geNorm and NormFinder and therefore was omitted by BestKeeper from further analysis due to high SD value. This indicated the similarities in results of different algorithms; nevertheless, the ranking of other genes is variable (Table 4). In order to overcome the discrepancies among different algorithms, rank aggregation was used. Our final list of genes by weighted rank aggregation showed that Hmbs and Ppia stayed on top of the list as suitable RGs, and HPRT was identified as a less stable RG in the hypothalamus (Table 4).

The study condition and panel of tested RGs have a great impact on the selection of suitable RG for normalization of data in qPCR. In hypothalamus of rat model of intermediate hypoxia, for example, GAPDH, B2m, ACTB and HPRT were identified as optimal RGs, while 18s was the least stable gene [42]. Selection of B2m, GAPDH and ACTB among constant RGs is similar to our study however, in current study, HPRT was the least stable gene in the hypothalamus. In another report, in acute toxicity studies, GAPDH and Phosphoglycerate Kinase 1 (Pgk1) were used as RGs in hypothalamus [43]; in which RGs were selected following monitoring of eighteen genes in liver and then four most stable genes from liver examined to select the suitable RG in hypothalamus. This indicates that even in same tissue, expression of common RGs varies in response to different study conditions, and emphasize on evaluating the expression stability in each study condition.

Analysis of expression of RGs in kidney showed different ranking by 3 different programs (Table 4); however, the results were quite similar. According to geNorm (Fig 3), M-values of B2m was more than 1.5, which indicates it is not a suitable RG in this organ. In addition, Bestkeeper omitted HPRT and B2m from further analysis due to high SD value (Table 3). The final ranking of 6 tested RGs in the kidney by weighted rank aggregation (Table 4) identified Hmbs, GAPDH,Ppia, and ACTB as the most stable genes while B2m is the most variable gene among the tested genes under the influence of testosterone. Similar to our study, GAPDH and ACTB were identified among optimal RGs in kidney in obese and lean Zuker rats in fasting and hyperglycemic models [44]. Ppia, was optimal candidate (along with PRS13) in clear cell renal carcinoma as compared to classical genes such as ACTB, GAPDH, 18s or B2m [45]. In current study, Ppia was among the suitable RGs in the kidneys. Another study in cattle kidney indicates out of six genes, GAPDH and Tyrosine 3-Monooxygenase/Tryptophan 5-Monooxygenase Activation Protein Zeta (YWHAZ) were identified to be the stable candidates [46]. These together, indicate that stable RG is dependent on tissue, experimental design and panel of tested genes.

Study in human muscle biopsy following testosterone therapy indicated that Ppia, Polymerase (RNA) II Subunit A (POLR2A) and Importin 8 (IPO8) were identified as suitable RG [20]. Even though HPRT is a variable gene among the examined set of genes under the influence of testosterone, unpublished data from our lab have identified GAPDH and HPRT as suitable RG in the uterus under the influence of estradiol in mature WKY rat. Another study by Das et al [33], showed that of hypophysectomized Sprague-Dawley rats, expression of Ppia, HPRT & GAPDH in the liver was not affected by gender. HPRT was reported as an optimal gene across various rat tissues, while B2m was less stable [9]. In another study, B2m, ACTB & HPRT were identified as suitable genes in the mammary gland during the rat estrous cycle [13]. In the brain of female Rhesus monkey, GAPDH was among the recommended genes for GABAergic system [8]. In our study, HPRT was less stable in both tissues i.e. kidney and hypothalamus, however B2m was among the stable genes in hypothalamus but not in kidney. Application of common RGs such as ACTB and GAPDH was tissue dependent even under same studied condition. In hypothalamus, GAPDH and ACTB were ranked as 4^th^ and 5^th^ stable genes, whilst in kidney, they were as ranked 2nd and 4^th^ stable genes.

Despites of all differences in the selection of suitable candidate gene in various experimental model, some researchers have only picked one classical housekeeping genes such as GAPDH, and ACTB [19,47–50]. Although historically RGs such as GAPDH & ACTB might not be suitable for all experimental conditions, optimal RGs must therefore be evaluated from a set of genes for each experimental condition such as specific treatment, different gender and tissue which might lead to identification of different RG.

There are some reports indicating that RG might participate in cell metabolisms and therefore, their level of expression could change. For example, in diabetic kidney, B2m contributes to diabetic tubulopathy and might not be a good RG for the kidney [51]. GAPDH has been reported to contribute to neurological disorder such as Huntington and Alzheimer diseases [52] which indicates GAPDH might not be an optimal candidate gene in hypothalamus. In addition, differences in signaling pathway might influence the result, for instance androgen receptor expression in hypothalamus is relatively high, whereas in kidney, this receptor showed very weak expression [53,54]. These might affect various signaling pathways under the testosterone influence [55].

In summary, an optimal RG should be a gene with minimal variable expression level under different conditions in a given tissue, the characteristic that makes suitable control for normalization of qPCR data. This report underlines the necessity of rigorous selection of RGs to ensure the reliability of the normalization process in qPCR for each tissue. Furthermore, with the employment of weighted rank aggregation, the results of different validated program could be combined together to produce one final list and overcomes the discrepancies among different algorithms.

The current study has various limitations which could affect the results, with the main one being the number of investigated genes. In a systematic review, Chapman *et al* [56] suggested suitable RG should be chosen from the panel of 10–20 genes. In addition, we did not include tissue specific RG, although we selected the most stable genes based on the current panel to enhance accuracy of data analysis. Furthermore, we did not include any tissue specific genes to ensure exact tissue collection, particularly the hypothalamus.

## Supporting information

S1 Table Ct values in hypothalamus. (DOCX)

S2 Table Ct values in kidney. (DOCX)

S1 File R script for rank aggregation. (DOCX)
