# Supplementary material for: Selection of suitable endogenous reference genes for qPCR in kidney and hypothalamus of rats under testosterone influence
Source: PLoS One. 2017 Jun 7;12(6):e0176368. doi: 10.1371/journal.pone.0176368 (PMC5462341; doi:10.1371/journal.pone.0176368)
Supplement: S1 Table — (DOCX) [file pone.0176368.s001.docx]

| Table S1- Hypothalamus Ct | | | | | | |
| --- | --- | --- | --- | --- | --- | --- |
| Sample Name | GAPDH | ACTB | Ppia | Hmbs | B2m | HPRT |
| SHAM1 | 22.70 | 21.97 | 22.57 | 29.57 | 23.81 | 25.35 |
| SHAM2 | 22.70 | 22.02 | 22.22 | 29.53 | 22.87 | 25.37 |
| SHAM3 | 22.86 | 22.09 | 22.84 | 29.40 | 23.64 | 25.26 |
| SHAM1 | 21.80 | 22.53 | 22.95 | 30.25 | 23.74 | 25.82 |
| SHAM2 | 21.92 | 22.63 | 22.40 | 29.09 | 23.09 | 25.69 |
| SHAM3 | 21.98 | 22.40 | 22.78 | 29.21 | 24.49 | 25.85 |
| SHAM1 | 22.45 | 22.33 | 22.59 | 30.29 | 23.80 | 25.82 |
| SHAM2 | 22.36 | 22.57 | 22.79 | 29.27 | 22.86 | 25.74 |
| SHAM3 | 21.59 | 22.40 | 22.23 | 28.88 | 23.75 | 25.79 |
| SHAM1 | 21.87 | 22.46 | 22.77 | 29.19 | 23.12 | 25.35 |
| SHAM2 | 21.87 | 22.48 | 22.39 | 29.82 | 23.46 | 25.61 |
| SHAM3 | 22.05 | 22.49 | 22.49 | 29.63 | 23.06 | 25.27 |
| ORX1 | 22.92 | 22.33 | 22.62 | 30.67 | 24.05 | 26.42 |
| ORX2 | 22.96 | 22.60 | 22.12 | 30.44 | 24.12 | 25.69 |
| ORX3 | 22.86 | 22.81 | 22.10 | 30.30 | 24.07 | 25.61 |
| ORX1 | 22.92 | 22.53 | 21.56 | 30.38 | 23.35 | 25.90 |
| ORX2 | 23.03 | 22.29 | 23.16 | 30.44 | 23.85 | 25.67 |
| ORX3 | 22.96 | 22.66 | 22.22 | 30.31 | 23.68 | 25.83 |
| ORX1 | 22.99 | 22.67 | 23.39 | 30.43 | 24.11 | 25.60 |
| ORX2 | 23.09 | 21.93 | 21.88 | 30.30 | 24.04 | 26.12 |
| ORX3 | 23.03 | 22.53 | 22.86 | 30.46 | 23.93 | 25.32 |
| ORX1 | 22.96 | 21.91 | 22.59 | 30.34 | 23.42 | 25.93 |
| ORX2 | 22.87 | 21.86 | 22.12 | 30.55 | 23.44 | 25.91 |
| ORX3 | 22.85 | 22.73 | 22.45 | 30.47 | 23.75 | 25.75 |
| ORX125-1 | 22.43 | 22.82 | 23.74 | 30.12 | 23.52 | 26.36 |
| ORX125-2 | 22.41 | 23.79 | 22.69 | 30.07 | 23.97 | 26.50 |
| ORX125-3 | 22.41 | 23.80 | 23.00 | 30.04 | 23.67 | 27.39 |
| ORX125-1 | 22.40 | 23.40 | 23.17 | 29.92 | 23.48 | 27.17 |
| ORX125-2 | 22.42 | 22.94 | 23.27 | 30.04 | 23.54 | 27.07 |
| ORX125-3 | 22.41 | 23.20 | 23.09 | 30.12 | 22.98 | 26.92 |
| ORX125-1 | 22.79 | 23.71 | 22.62 | 30.09 | 23.96 | 26.85 |
| ORX125-2 | 22.43 | 23.13 | 22.92 | 30.10 | 23.84 | 25.91 |
| ORX125-3 | 22.41 | 23.51 | 23.14 | 30.20 | 24.37 | 26.80 |
| ORX125-1 | 22.40 | 23.29 | 23.23 | 30.13 | 22.95 | 26.94 |
| ORX125-2 | 22.42 | 23.53 | 22.75 | 30.33 | 23.50 | 27.55 |
| ORX125-3 | 22.39 | 24.12 | 23.08 | 29.96 | 23.41 | 27.04 |
| ORX250-1 | 22.54 | 22.72 | 23.54 | 30.42 | 23.78 | 28.48 |
| ORX250-2 | 22.38 | 21.59 | 23.29 | 30.63 | 23.55 | 28.80 |
| ORX250-3 | 22.28 | 22.60 | 23.86 | 29.64 | 23.77 | 28.56 |
| ORX250-1 | 22.30 | 22.47 | 23.38 | 30.44 | 24.00 | 28.42 |
| ORX250-2 | 21.81 | 22.89 | 23.47 | 30.50 | 23.72 | 28.41 |
| ORX250-3 | 22.47 | 22.73 | 23.44 | 30.49 | 23.54 | 28.65 |
| ORX250-1 | 22.55 | 22.47 | 23.62 | 31.06 | 24.09 | 28.63 |
| ORX250-2 | 23.04 | 22.70 | 23.53 | 30.61 | 23.65 | 28.80 |
| ORX250-3 | 22.16 | 23.32 | 23.84 | 30.90 | 23.25 | 29.03 |
| ORX250-1 | 22.41 | 22.84 | 23.58 | 30.48 | 24.03 | 28.03 |
| ORX250-2 | 22.20 | 22.87 | 23.75 | 29.99 | 24.42 | 27.94 |
| ORX250-3 | 21.79 | 22.08 | 23.94 | 30.08 | 24.08 | 27.68 |
