# Supplementary material for: Selection of suitable endogenous reference genes for qPCR in kidney and hypothalamus of rats under testosterone influence
Source: PLoS One. 2017 Jun 7;12(6):e0176368. doi: 10.1371/journal.pone.0176368 (PMC5462341; doi:10.1371/journal.pone.0176368)
Supplement: S2 Table — (DOCX) [file pone.0176368.s002.docx]

| Table S2- Kidney Ct value | | | | | | |
| --- | --- | --- | --- | --- | --- | --- |
| Sample Name | GAPDH | ACTB | Ppia | Hmbs | B2m | HPRT |
| SHAM1 | 22.81 | 21.99 | 23.14 | 30.86 | 27.34 | 33.40 |
| SHAM2 | 22.78 | 22.14 | 23.51 | 30.97 | 27.47 | 33.58 |
| SHAM3 | 22.91 | 21.72 | 23.66 | 30.94 | 27.73 | 33.81 |
| SHAM1 | 22.88 | 21.62 | 23.18 | 30.94 | 27.51 | 32.80 |
| SHAM2 | 22.85 | 21.76 | 23.21 | 30.67 | 27.91 | 33.33 |
| SHAM3 | 22.96 | 22.34 | 23.02 | 30.50 | 27.74 | 33.07 |
| SHAM1 | 22.24 | 21.76 | 23.93 | 30.90 | 26.92 | 33.66 |
| SHAM2 | 22.62 | 21.90 | 23.69 | 31.05 | 27.08 | 33.39 |
| SHAM3 | 22.60 | 21.21 | 24.06 | 30.68 | 26.83 | 33.71 |
| SHAM1 | 23.07 | 21.58 | 23.52 | 30.98 | 27.22 | 33.14 |
| SHAM2 | 22.83 | 21.87 | 23.62 | 30.73 | 27.38 | 33.10 |
| SHAM3 | 23.27 | 21.58 | 23.77 | 30.80 | 27.12 | 33.07 |
| ORX1 | 23.35 | 22.86 | 23.19 | 30.01 | 23.67 | 27.38 |
| ORX2 | 22.79 | 22.49 | 23.44 | 30.01 | 23.86 | 27.24 |
| ORX3 | 23.28 | 22.82 | 23.08 | 29.99 | 23.95 | 27.41 |
| ORX1 | 23.22 | 22.80 | 22.73 | 30.13 | 23.92 | 27.36 |
| ORX2 | 23.26 | 22.64 | 22.80 | 30.02 | 23.71 | 27.33 |
| ORX3 | 23.12 | 22.89 | 22.70 | 30.38 | 23.88 | 27.26 |
| ORX1 | 23.82 | 22.65 | 23.20 | 29.76 | 23.48 | 27.38 |
| ORX2 | 23.88 | 22.60 | 23.30 | 30.16 | 23.88 | 27.33 |
| ORX3 | 23.53 | 22.54 | 22.91 | 30.05 | 23.71 | 27.18 |
| ORX1 | 23.50 | 22.58 | 23.16 | 30.25 | 23.69 | 27.25 |
| ORX2 | 23.12 | 22.45 | 23.14 | 30.23 | 23.81 | 27.26 |
| ORX3 | 23.19 | 22.67 | 23.10 | 29.87 | 23.88 | 29.35 |
| ORX125-1 | 22.83 | 21.95 | 21.84 | 30.86 | 30.71 | 29.42 |
| ORX125-2 | 22.60 | 22.38 | 21.60 | 30.59 | 31.13 | 29.27 |
| ORX125-3 | 22.60 | 22.35 | 21.82 | 30.77 | 30.78 | 29.55 |
| ORX125-1 | 22.35 | 22.34 | 21.59 | 30.84 | 30.66 | 29.81 |
| ORX125-2 | 22.78 | 22.68 | 21.52 | 31.00 | 30.92 | 29.91 |
| ORX125-3 | 22.88 | 22.32 | 21.63 | 30.60 | 30.99 | 29.89 |
| ORX125-1 | 22.32 | 22.66 | 21.67 | 30.92 | 31.32 | 29.66 |
| ORX125-2 | 22.31 | 22.56 | 21.60 | 30.66 | 31.25 | 29.97 |
| ORX125-3 | 21.94 | 22.40 | 21.60 | 30.74 | 31.55 | 29.77 |
| ORX125-1 | 22.01 | 21.99 | 21.36 | 30.89 | 30.35 | 29.15 |
| ORX125-2 | 22.50 | 21.70 | 21.50 | 30.89 | 29.82 | 29.16 |
| ORX125-3 | 22.53 | 21.89 | 21.39 | 30.62 | 30.07 | 29.12 |
| ORX250-1 | 22.94 | 22.40 | 21.79 | 30.08 | 22.13 | 28.05 |
| ORX250-2 | 22.73 | 22.28 | 21.74 | 29.78 | 22.15 | 29.40 |
| ORX250-3 | 22.51 | 22.52 | 21.29 | 29.93 | 22.50 | 28.04 |
| ORX250-1 | 22.53 | 22.57 | 23.58 | 30.04 | 21.84 | 28.55 |
| ORX250-2 | 22.43 | 22.90 | 23.89 | 30.39 | 22.24 | 28.96 |
| ORX250-3 | 22.75 | 22.59 | 23.65 | 30.10 | 22.37 | 28.75 |
| ORX250-1 | 22.66 | 22.39 | 22.65 | 30.01 | 22.30 | 28.85 |
| ORX250-2 | 22.71 | 22.34 | 22.56 | 29.99 | 22.48 | 28.94 |
| ORX250-3 | 22.80 | 22.77 | 22.26 | 30.21 | 22.90 | 28.58 |
| ORX250-1 | 22.77 | 22.25 | 22.44 | 29.87 | 22.14 | 28.43 |
| ORX250-2 | 22.66 | 22.54 | 22.49 | 29.75 | 21.97 | 28.12 |
| ORX250-3 | 22.54 | 22.50 | 21.98 | 29.97 | 22.13 | 28.14 |
