# Supplementary material for: Selection of suitable endogenous reference genes for qPCR in kidney and hypothalamus of rats under testosterone influence
Source: PLoS One. 2017 Jun 7;12(6):e0176368. doi: 10.1371/journal.pone.0176368 (PMC5462341; doi:10.1371/journal.pone.0176368)
Supplement: S1 File — (DOCX) [file pone.0176368.s003.docx]

**HYPOTHALAMUS:**

> library(rstudio)

> library(RankAggreg)

> library(gtools)

> x2 <- matrix(c("HMBS", "GAPDH", "B2M", "PPIA", "ACTB","HPRT",

+ "HMBS", "PPIA", "B2M", "GAPDH", "ACTB","HPRT",

+ "PPIA", "HMBS", "ACTB", "B2M", "GAPDH","HPRT"), byrow=T, ncol=6)

> w2<-matrix(c(.45, .45, .5, .59, .63, .79,

+ .31, .37, .43, .55, .55, 1.04,

+ .74, .66, .46, .38, .1, .87), byrow=T, ncol=6)

> w2 <- t(apply(w2, 1, sort))

> (toplist2 <- BruteAggreg(x2,6,w2,"Spearman")) # using the Spearman distance

The optimal list is:

PPIA HMBS B2M GAPDH ACTB HPRT

Algorithm: BruteForce

Distance: Spearman

Score: 1.104705

**KIDNEY:**

>plot(toplist2)

> library(rstudio)

> library(RankAggreg)

> library(gtools)

> x2 <- matrix(c("ACTB", "GAPDH", "HMBS", "PPIA", "HPRT","B2M",

+ "HMBS", "GAPDH", "ACTB", "PPIA", "HPRT","B2M",

+ "HMBS", "PPIA", "GAPDH", "ACTB", "B2M","HPRT"), byrow=T, ncol=6)

> w2<-matrix(c(.5, .5, .64, .77, 1.32, 1.96,

+ .34, .89, 1.07, 1.08, 1.86, 3.21,

+ .91, .12, -.24, -.62, .84, .82), byrow=T, ncol=6)

> w2 <- t(apply(w2, 1, sort))

> (toplist2 <- BruteAggreg(x2,6,w2,"Spearman")) # using the Spearman distance

The optimal list is:

HMBS GAPDH PPIA ACTB HPRT B2M

Algorithm: BruteForce

Distance: Spearman

Score: 0.4682255
